# Boundary-Preserved Deep Denoising of Stochastic Resonance Enhanced Multiphoton Images

**DOI:** 10.1109/JTEHM.2022.3206488

**Published:** 2022-09-14

**Authors:** Sheng-Yong Niu, Lun-Zhang Guo, Yue Li, Zhiming Zhang, Tzung-Dau Wang, Kai-Chun Liu, You-Jin Li, Yu Tsao, Tzu-Ming Liu

**Affiliations:** Research Center for Information Technology Innovation (CITI)Academia Sinica 38017 Taipei 11529 Taiwan; Department of Computer Science and EngineeringUniversity of California San Diego 8784 San Diego CA 92093 USA; Department of Biomedical EngineeringNational Taiwan University 33561 Taipei 10617 Taiwan; Institute of Translational Medicine, Faculty of Health Sciences & Ministry of Education Frontiers Science Center for Precision Oncology, University of Macau, Taipa 59193 Macau China; Cardiovascular Center and Division of CardiologyDepartment of Internal MedicineCollege of Medicine, National Taiwan University Hospital 38006 Taipei 10002 Taiwan; Department of Electrical EngineeringChung Yuan Christian University 34900 Taoyuan 32023 Taiwan

**Keywords:** Third harmonic generation, three-photon fluorescence, deep denoising autoencoder

## Abstract

Objective: With the rapid growth of high-speed deep-tissue imaging in biomedical research, there is an urgent need to develop a robust and effective denoising method to retain morphological features for further texture analysis and segmentation. Conventional denoising filters and models can easily suppress the perturbative noise in high-contrast images; however, for low photon budget multiphoton images, a high detector gain will not only boost the signals but also bring significant background noise. In such a stochastic resonance imaging regime, subthreshold signals may be detectable with the help of noise, meaning that a denoising filter capable of removing noise without sacrificing important cellular features, such as cell boundaries, is desirable. Method: We propose a convolutional neural network-based denoising autoencoder method — a fully convolutional deep denoising autoencoder (DDAE) — to improve the quality of three-photon fluorescence (3PF) and third-harmonic generation (THG) microscopy images. Results: The average of 200 acquired images of a given location served as the low-noise answer for the DDAE training. Compared with other conventional denoising methods, our DDAE model shows a better signal-to-noise ratio (28.86 and 21.66 for 3PF and THG, respectively), structural similarity (0.89 and 0.70 for 3PF and THG, respectively), and preservation of the nuclear or cellular boundaries (F1-score of 0.662 and 0.736 for 3PF and THG, respectively). It shows that DDAE is a better trade-off approach between structural similarity and preserving signal regions. Conclusions: The results of this study validate the effectiveness of the DDAE system in boundary-preserved image denoising. Clinical Impact: The proposed deep denoising system can enhance the quality of microscopic images and effectively support clinical evaluation and assessment.

## I. Introduction

Real-time visualization of living cells in their tissue environment is crucial for many applications in life sciences and medical devices [1], [2]. For instance, deep-tissue cellular imaging with transgenic labeling of reporters could reveal niche environments and functional interactions of multiple cells in the context of hematopoiesis [3], tumor metastasis [4], [5], [6], and neuronal connection [7], [8]. *In vitro* or non-invasive *in vivo* imaging flow cytometry (IFC) through real-time microscopy of the intra-fluidic channel or intravascular blood cells could have the potential to benefit patients and caregivers in detecting physiological aberrancy more efficiently [9], [10], [11], [12], [13]. Several studies have successfully employed IFC to provide quantitative image data of cellular targets to support decision-making in clinical diagnosis [14]. Results show that IFC can significantly improve the classification accuracy of routine white blood cell counts by their sizes, shapes, structures, and nucleus-to-cytoplasm ratios.

To obtain sharp sectioning images of cells in tissues, researchers developed confocal or multiphoton fluorescence microscopy to improve axial resolution [1], [15]. For instance, two-photon fluorescence microscopy has been widely applied in many deep-tissue studies, such as in brain research. Its near-infrared (800–1300 nm) laser excitation can greatly reduce the photobleaching of probes, distortion of the wavefront, scattering of photons, and maintenance of the subcellular resolution of images [1], [16], [17]. For sufficient contrast of fluorescence imaging in deep tissues, the excitation intensity must be high, which can lead to background interference originating from the diffused fluorescence photons caused by multiple scattering. To obtain high-acuity images at depths greater than 700 $\mu \text{m}$, three-photon contrasts were excited by a 1700-nm high pulse energy laser. Three-photon microscopy can significantly improve emission localization, reducing the out-of-focus background compared to two-photon microscopy [18]. Various existing fluorescent dyes can still be used in three-photon fluorescence (3PF) microscopy. However, to realize in vivo imaging and medical testing such as virtual optical biopsy of immune cells, in vivo label-free microscopy is critical. Many researchers have focused on the development of third-harmonic generation (THG) microscopy for label-free imaging of tissues [11], [19], [20], [21], [22]. At an excitation wavelength of 1230 nm, THG microscopy can noninvasively obtain cellular morphology and visualize subcellular organelles in deep tissues without labeling. It can deliver an alternative contrast modality to complement multiphoton fluorescence microscopy, which provides information on cellular morphologies in three-dimensional tissue culture [17], [23].

Although there have been several studies on both 3PF and THG microscopies, their low signal-to-noise ratio remains a crucial issue for the delineation and segmentation of cells. The signal counts of third-order nonlinear optical microscopy are low and comparable to the noise counts resulting from the high bias voltages of the detection units. The type of noise includes signal-dependent Poisson noise and detector-dependent Gaussian noise. The former involves a random process of photon emission and the discrete nature of the photo-excited charges. The latter typically results from flicker or thermal noise in electronic systems. Under such low photon-budget conditions, the signals may have stochastic resonance effects, where sub-threshold signals can be boosted over the threshold with the help of detector noise [24]. The signal pattern within cells carries features of noise, making it difficult to extract the true signals from the background. Therefore, it is crucial to find an effective noise-filtering method to enhance image contrast while retaining structural information for further segmentation and texture analysis. Typically, researchers have applied Gaussian and median filters to remove Poisson or Gaussian noise. Poisson noise can be transformed by a stabilizing method such as Anscombe transformation into Gaussian white noise and a Gaussian noise filter can be utilized to alleviate it [25]. The nonlocal mean method performs noise filtering on image patches with similar textures. Block-matching and collaborative filtering methods, such as block-matching and 3D filtering (BM3D), have been proposed and widely used [26]. The feasibility of these enhancement approaches has been validated in various clinical examinations, including breast cancer [27], pancreatic neuroendocrine tumors [28], and hematology [29]. In addition, some researchers have used the Bayesian method to extract noise information from prior knowledge of images, train the likelihood function to predict the residual images V, and remove them from noisy observations [25], [30]. These methods perform well in many image-denoising problems; however, conventional signal processing assumes that noise is a linear addition of signals by $y = S+V$, where $S$ represents the signals and $V$ represents the noise. This model is appropriate when noise $V$ is perturbative to $S$; however, in the stochastic resonance regime, $V$ is comparable to or larger than $S$ and the detection threshold $T$ is larger than $S$ in many-pixel images. The representation of overall signals should be $y=S+V-T\,\,\mathrm {if }\,\,S+V>T\mathrm {;0,\,\,if}\,\,S+V < T$. Therefore, the conventional de-speckle filter, median filter, or Gaussian filter may decrease the details of cellular morphology. For signal-noise entangled stochastic resonance images, these methods may not work well because of the difference in noise modeling, that is, the noisy observation is not simply a superposition of signals and noise. Hence, for low photon-budget multiphoton microscopy images acquired in high-speed or deep-tissue imaging, it remains a challenging task to enhance the signal-to-noise ratio without sacrificing structural information.

Several machine learning and deep learning-based denoising methods have been proposed and demonstrated better filtering results compared to traditional filters. In particular, deep convolutional neural network (CNN)-based algorithms have been widely applied in image classification [30], segmentation [31], and denoising [32], [33]. In the field of acoustics, researchers have proposed CNN-based methods and denoising autoencoder (DAE) architectures to perform speech denoising [34], [35], [36], where the DAE method successfully filtered background noise and improved the perceptual evaluation of speech quality (PESQ). Reference [33]. Inspired by the success of CNN and DAE methods, in this work we propose a fully convolutional deep denoising autoencoder (DDAE) method to reduce noise in low photon budget multiphoton microscopy images, especially for 3PF and THG images. In our experiments, we confirm that the DDAE model outperforms the Gaussian filter, median filter, and the benchmark BM3D algorithm in terms of signal-to-noise ratio and structural similarity. Moreover, DDAE effectively preserves stochastic resonance-enhanced features so that regions of nuclei or cells can be delineated more correctly, which is important for further segmentation.

## II. Methods

### A. Cell Culture, Cell Staining, and Acquisition of Multiphoton Images

RAW 264.7 — a murine macrophage cell line — was plated on bottom glass dishes (Nest Scientific, 801001) and cultured in Dulbecco’s modified Eagle’s medium (DMEM) containing 10% fetal bovine serum (FBS), 100 U/ml penicillin, and 100 $\mu \text{g}$/ml streptomycin. For 3PF imaging, no further treatment was added. For THG imaging, three hours after plating, 50 ng/ml lipopolysaccharide (LPS; Sigma-Aldrich) was used to elicit inflammatory macrophages in the M1 state. After 24 h of cytokine stimulation, the medium was replaced with a normal medium for cell imaging. For 3PF imaging, $2~\mu \text{g}$/mL Hoechst 33342 (Thermo Fisher Scientific) was used to stain cell nuclei for 5 min. Unloaded Hoechst 33342 was removed by washing the cells with normal medium. For THG imaging, there was no cell labeling. Lipid granules in M1-activated macrophages can produce strong THG signals.

Time-lapse 3PF and THG images were acquired using an inverted multiphoton microscope (A1MP^+^; Nikon, Japan) and a near-infrared (800–1300 nm) femtosecond laser (InSight X3, Spectra-Physics, Mountain View, California) with a 100-fs pulse width and 80-MHz repetition rate was used as the excitation source. The operation wavelength for the 3PF and THG images was 1250 nm, which has the least on-focus phototoxicity and deepest penetration depth for biomedical samples. The laser light first transmitted an 820 nm edged the multiphoton dichroic beam splitter and was then focused through a water-immersed $40\times $ and 1.15 NA objective. To avoid photobleaching, the Hoechst blue-labeled cells were excited at an average power of 11 mW (100 GW/cm^2^ instantaneous intensity), whereas 37 mW (335 GW/cm^2^ instantaneous intensity) was required to obtain detectable signals. All the pairs of the resonant scanner and galvanometer mirrors generated three-photon signals that were epi-collected by the same objective, reflected by the 820 nm edged multiphoton dichroic beam splitter, which was further reflected by a 495-nm edged dichroic beam splitter in the non-descanned detection unit, filtered by a 415–485 nm bandpass filter, and finally detected by the same photomultiplier tubes. Then, the laser was raster scanned to perform point-by-point excitation and detection, forming 512 $\times512$ pixel images at a 15-Hz frame rate. All the images were subsequently exported to the TIFF format for denoising and deep learning processes.

### B. Traditional Denoising Methods

For the Gaussian and median filters, we used the Python SciPy function ndimage.gaussian_filter and ndimage.median_filter to perform Gaussian and median filtering with standard deviations $\sigma $ (sigma values) of 1, 3, 5, and 10. For the BM3D method, we implemented MATLAB codes from https://www.cs.tut.fi/~foi/GCF-BM3D/ to perform BM3D denoising with noise standard deviations $\sigma $ (sigma values) of 120, 140, 160, 180, 200, 220, and 240.

### C. Fully Convolutional Deep Denoising Autoencoder Model

We used the Keras framework to implement the DDAE model with a fully convolutional neural network architecture. A 5-fold cross-validation method was used to validate the proposed DDAE model. We divided all data into five groups. One group was considered as the testing set, and the remaining four groups were used for training. During training, we randomly selected one group as the validation set, while the other three groups were used to train the DDAE models. The cross-validation approach repeated five iterations until all groups have been tested. Next, we repeated the 5-fold cross-validation three times, with random selection of grouped data for each round. The final reported average results across all folds from all runs were used to assess the reliability of the proposed DDAE.

We trained the model with 50 epochs for the THD and 3PF, and early stopping with the patience of 10 epochs was applied for model training to avoid overfitting. The loss for training DDAE was the mean-squared error. The architecture of DDAE and its details are shown in Table 1. As shown in the table, different numbers of filters are investigated in this work.

**TABLE 1:** Architecture Details of DDAE

DDAE	Layer Information	
Encoder	Layer 1	Conv2D, $f\mathrm {=m}$, $k\mathrm {=3\times 3}$, ReLU, MaxPooling2D $k\mathrm {=2\times 2}$
	Layer 2	Conv2D, $f\mathrm {=m\times 2}$, $k\mathrm {=3\times 3}$, ReLU, MaxPooling2D $k\mathrm {=2\times 2}$
Bottleneck Layer	Layer 3	Conv2D, $f\mathrm {=m\times }4$, $k\mathrm {=3\times 3}$, RELU,
Decoder	Layer 4	Conv2D, $f\mathrm {=m\times 4}$, $k\mathrm {=3\times 3}$, ReLU, UpSampling2D $k\mathrm {=2\times 2}$, ReLU
	Layer 5	Conv2D, $f\mathrm {=m\times 2}$, $k\mathrm {=3\times 3}$, ReLU, UpSampling2D $k\mathrm {=2\times 2}$, ReLU
Output Layer	Layer 6	Conv2D, $f=1$, $k\mathrm {=3\times 3}$, sigmoid

Note: $f$ and $k$ denote the number of filters and the filter size, respectively, and $\mathrm {m=8, 16, and 32}$

### D. Analyzing the Contrast and Structural Similarity of Restored Images

To evaluate the signal-to-noise ratio and the restoration of structural information, we used peak signal-to-noise ratio (PSNR) analysis [37] and the structural similarity index (SSIM) [38].

### E. Analyzing the Restoration of Nuclear and Cellular Boundaries

**FIGURE 1.**
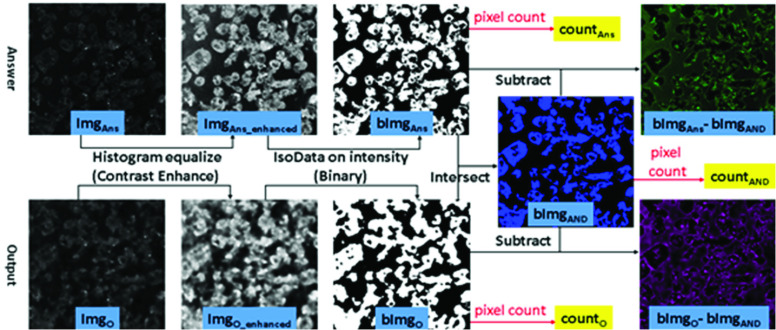
The procedures of image processing for analyzing the preservation of nuclear and cellular boundaries.

**FIGURE 2.**
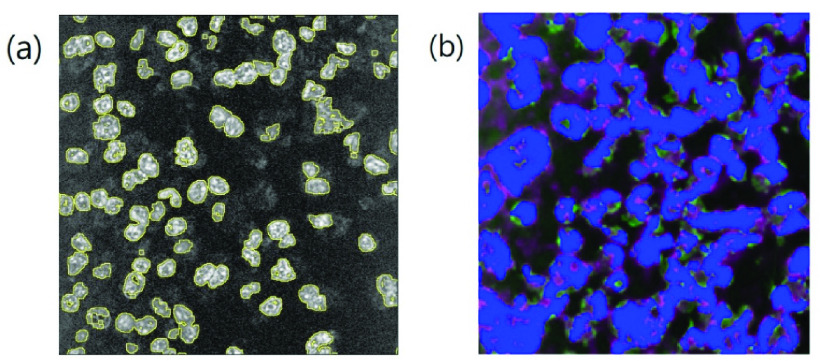
(a) The boundaries (yellow contours) of the binarized 3PF answer image bImag $_{\mathbf {Ans}}$ can precisely outline the nuclear boundaries. (b) The superposition image of the true positive (blue color), the false positive (magenta color), and the false negative (green color) parts of denoised THG images. Fields of view: (a) $120\times 120\,\,\mu \text{m}$; (b) $160\times 160\,\,\mu \text{m}$.

To assess how well the denoising filter retained the stochastic resonance-enhanced features in cells, we analyzed the precision, recall, specificity and F-measure of nuclear and cellular boundaries from the denoised image **Img** $_{\mathbf {O}}$ and the low-noise answer image **Img** $_{\mathbf {Ans}}$. The contrasts of **Img** $_{\mathbf {O}}$ and **Img** $_{\mathbf {Ans}}$ were first enhanced through histogram equalization (see Fig. 1), and then binarized using the intensity auto-threshold method — IsoData — thereby obtaining the binary images of **bImg** $_{\mathbf {O}}$ and **bImg** $_{\mathbf {Ans}}$, respectively. The boundaries of the binarized answer images could precisely depict the boundaries of the cells and nuclei [Fig. 2(a)]. **bImg** $_{\mathbf {AND}}$ is the overlap of **bImg** $_{\mathbf {O}}$ and **bImg** $_{\mathbf {Ans}}$, representing the true positive of the nucleus (in the 3PF images) or cell (in the THG images) regions. We also use a NOT OR gate to the **bImg** $_{\mathbf {O}}$ and **bImg** $_{\mathbf {Ans}}$ to obtain the true negative of the nucleus **bImg** $_{\mathbf {NOR,}}$ and inverse bImgAns (as **bImg** $_{\mathbf {IAns}}$**)** to represent the actual negative. Finally, we used the pixel counts of the five binary images, **count** $_{\mathbf {O}}$, **count** $_{\mathbf {Ans}}$, **count** $_{\mathbf {AND}}$, **count** $_{\mathbf {NOR}}$, and **count** $_{\mathbf {IAns}}$ to compute the precision, recall, specificity and F-measure of the denoised images, using the following functions:

\begin{align*} \mathrm {precision}=&\frac {{\mathbf {count}}_{AND}}{{\mathbf {count}}_{\mathrm {O}}}, \tag{1}\\ \mathrm {recall}=&\frac {{\mathbf {count}}_{\mathrm {AND}}}{{\mathbf {count}}_{\mathrm {Ans}}}, \tag{2}\\ \mathrm {Specificity}=&\frac {{\mathbf {count}}_{\mathrm {NOR}}}{{\mathbf {count}}_{\mathrm {IAns}}}, \tag{3}\\ \mathrm {F-measure}=&\frac {\mathrm {2\ast precision\ast recall}}{\mathrm {precision+recall}}.\tag{4}\end{align*}

We also created pseudo-color images to visualize the **retained** regions of nuclei or cells (blue, Fig. 1), false negative regions of **bImg** $_{\mathbf {Ans}}$**- bImg** $_{\mathbf {AND}}$ (green, Fig. 1), and false positive regions of **bImg** $_{\mathbf {O}}$**- bImg** $_{\mathbf {AND}}$ (magenta, Fig. 1). We then combined these three pseudo-color images to obtain the superposition image [Fig. 2(b)], such that the mismatch of boundaries could be visualized.

## III. Results

To evaluate the denoising performance of the different approaches, we performed 1250 nm excited 3PF and THG microscopy on RAW 264.7. The 3PF contrast mostly labeled the nuclei, and the THG contrast revealed lipid granules within cells. The excitation intensity used for the 3PF microscopy was too low to generate sufficient THG signals and for 3PF imaging, we did not activate the proliferation of lipid granules in the RAW cells. There was no crosstalk between the 3PF and THG signals.

At each observation location in a petri-dish, we acquired 200 images at a 15-Hz frame rate, with fixed excitation power, using the same detection channel and bias voltage. The acquired images served as low-photon-budget images to be denoised. To evaluate the denoising performance, we obtained a low-noise answer image of the corresponding location from the average of the acquired 200 images. The low-noise answer image also served as a low-noise answer for DDAE training. Several studies have utilized a similar approach to obtain ground-truth images [39], [40], [41] and we referred to the same principles to select the required number of images for ground-truth estimation [42].

For each imaging modality, we selected 31 locations on each petri-dish and acquired 31 image batches. All the batches were used for 5-fold cross-validation to validate DDAE. Then, we compared the results with those processed by traditional denoising methods such as Gaussian filter, median filter, and BM3D. We measured the quality of the results using PSNR and SSIM to represent the fidelity of the signal and structures. BM3D. We measured the quality of results by PSNR and SSIM to represent the fidelity of signal and structures.

### A. Denoising of Three-Photon Fluorescence Images

**FIGURE 3.**
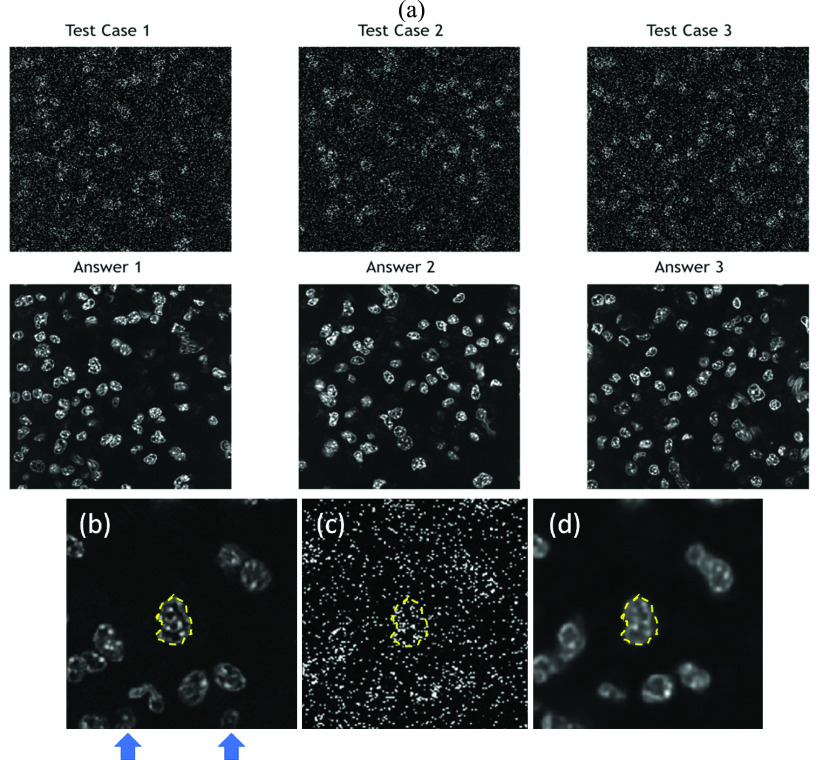
(a) Noisy inputs (upper rows) and low-noise answers (bottom rows) of three testing 3PF images of Hoechst blue labeled RAW cells. (b) The low-noise answer image, (c) noisy input, and (d) DDAE processed one. Processed by DDAE model, the noise was suppressed, the contrast was enhanced, and the nuclear boundary was well-preserved (yellow dashed closure). Fields of views: (a) $120\times 120\,\,\mu \text{m}$; (b–d) $50\times 50\,\,\mu \text{m}$.

**FIGURE 4.**
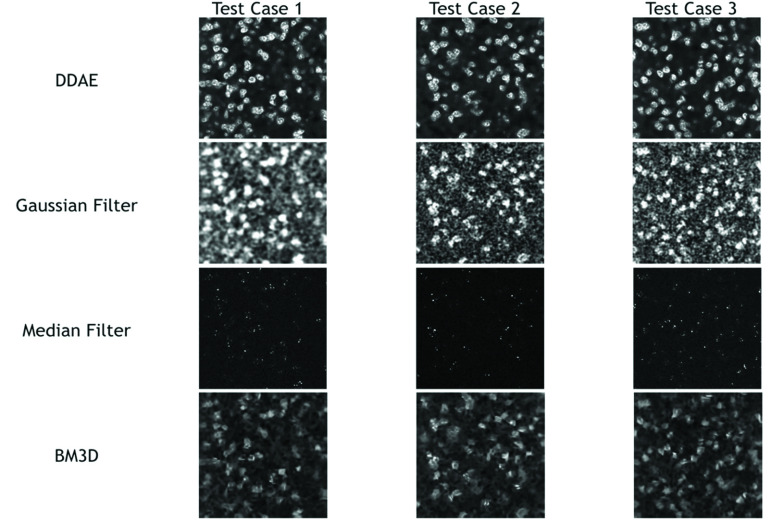
Denoising results of 3PF images with DDAE, Gaussian filter, median filter, and BM3D algorithms.

Three testing cases of 3PF images were sampled from each testing batch. Given that the images were acquired at a 1/15-s frame time, they contained a lot of salt-and-pepper noise [Fig. 3(a), upper row]. The stochastic resonance-enhanced signals made the cells faintly discernible [Fig. 3(c)]. By applying the trained DDAE model, they showed a great improvement in image contrast [Fig. 3(a), bottom row]. The nuclear boundaries were well preserved [Fig. 3(d), yellow-dashed contour]. For cells with relatively low signal levels in low-noise answer images [Fig. 3(b), indicated by blue arrows], noise cannot boost them in the high frame-rate image, and the DDAE cannot restore them in such a situation. Compared with traditional filtering methods such as the Gaussian filter, median filter, and BM3D (Fig. 4), the results of the 3PF DDAE denoising surpass all of them on the PSNR and SSIM scores (Table 2 and Appendix Table 4). In general, the DDAE method achieves a higher signal-to-noise ratio of 28.86 PSNR and retains more structural information of 0.89 SSIM. Among the traditional methods, by choosing the optimal sigma value, the performance of the BM3D filter is the best with 38.66 PSNR. It shows a relatively clear cellular outline but loses several intracellular details with 0.88 SSIM. As for the results of the median filter, although it can retain a few significant signal spots, it cannot show nuclear boundaries. The Gaussian filter method generally performs better than the median filter, but it contained significant noise and vague nuclear boundaries in the results. In brief, 3PF DDAE model can improve the image quality better than most traditional denoising methods, and users can obtain results in a few seconds without time-consuming steps, such as by trying optimal sigma values.

**TABLE 2:** Average PSNR and SSIM Scores Yielded by Different Denoising Approaches

Approach	3PF Images		THG Images	
	PSNR	SSIM	PSNR	SSIM
DDAE	28.86 ( $\text{k}=32$)	0.89( $\text{f}=32$)	21.66 ( $\text{k}=8$)	0.70 ( $\text{f}=16$)
Gaussian	28.01 ( $\sigma =5$)	0.87 ( $\sigma =5$)	16.6 ( $\sigma =3$)	0.52 ( $\sigma =10$)
Median	20.69 ( $\sigma =5$)	0.23 ( $\sigma =3$)	14.12 ( $\sigma =3$)	0.16 ( $\sigma =1$)
BM3D	38.66( $\sigma =120$)	0.88 ( $\sigma =120$)	26.38( $\sigma =120$)	0.41 ( $\sigma =120$)

**TABLE 3:** The Precision, Recall, and F1- Scores Yielded by Different Denoising Approaches

Approach	3PF Images				THG Images			
	Precision	Recall	Specificity	F1-score	Precision	Recall	Specificity	F1-score
DDAE8	0.589	0.766	0.940	0.662	0.663	0.832	0.853	0.736
DDAE16	0.592	0.769	0.941	0.667	0.663	0.832	0.852	0.736
DDAE32	0.581	0.783	0.937	0.664	0.659	0.838	0.848	0.736
BM3D120	0.460	0.867	0.837	0.586	0.712	0.704	0.887	0.694
BM3D140	0.446	0.869	0.819	0.571	0.734	0.651	0.900	0.669
BM3D160	0.460	0.848	0.826	0.573	0.761	0.585	0.919	0.635
BM3D180	0.479	0.824	0.849	0.582	0.780	0.531	0.930	0.597
BM3D200	0.482	0.816	0.860	0.582	0.788	0.497	0.934	0.561
BM3D220	0.480	0.813	0.866	0.579	0.810	0.441	0.946	0.520
BM3D240	0.504	0.782	0.886	0.588	0.830	0.390	0.959	0.480
Gaussian1	0.274	0.658	0.808	0.384	0.566	0.433	0.885	0.488
Gaussian3	0.464	0.848	0.890	0.596	0.681	0.751	0.879	0.714
Gaussian5	0.472	0.882	0.892	0.613	0.619	0.817	0.826	0.703
Gaussian10	0.345	0.913	0.810	0.500	0.533	0.870	0.742	0.659
Median1	0.271	0.217	0.936	0.239	0.569	0.110	0.972	0.183
Median3	0.758	0.084	0.997	0.152	0.915	0.018	0.999	0.036
Median5	0.870	0.103	0.973	0.091	0.947	0.004	0.999	0.008
Median10	0.332	0.786	0.652	0.355	0.943	0.037	0.998	0.070

Note: Numbers in the approach column represent the filter numbers and sigma values of the algorithm.

**TABLE 4:** Denoising Performance of Filters on 3PF Images

		Gaussian Filter			
		1 $^{\mathbf {st}}$5-fold	2 $^{\mathbf {nd}}$5-fold	3 $^{\mathbf {rd}}$5-fold	average
$\sigma =$ 1	PSNR	21.96	22.07	22.01	22.01
	SSIM	0.46	0.46	0.46	0.46
$\sigma =$ 3	PSNR	27.72	27.81	27.77	27.77
	SSIM	0.83	0.83	0.83	0.83
$\sigma =$ 5	PSNR	27.98	28.08	27.99	28.01
	SSIM	0.87	0.87	0.87	0.87
$\sigma =$ 10	PSNR	26.93	27.06	26.93	26.97
	SSIM	0.86	0.87	0.86	0.86
		Median Filter			
		1 $^{\mathbf {st}}$5-fold	2 $^{\mathbf {nd}}$5-fold	3 $^{\mathbf {rd}}$5-fold	average
$\sigma =$ 1	PSNR	12.73	12.86	12.76	12.78
	SSIM	0.10	0.11	0.11	0.11
$\sigma =$ 3	PSNR	20.26	20.41	20.34	20.34
	SSIM	0.23	0.23	0.23	0.23
$\sigma =$ 5	PSNR	20.62	20.75	20.70	20.69
	SSIM	0.21	0.21	0.21	0.21
$\sigma =$ 10	PSNR	20.43	20.55	20.50	20.49
	SSIM	0.20	0.20	0.21	0.20
		BM3D			
		1 $^{\mathbf {st}}$5-fold	2 $^{\mathbf {nd}}$5-fold	3 $^{\mathbf {rd}}$5-fold	average
$\sigma =$ 120	PSNR	38.59	38.75	38.63	38.66
	SSIM	0.88	0.88	0.88	0.88
$\sigma =$ 140	PSNR	38.13	38.25	38.16	38.18
	SSIM	0.86	0.85	0.85	0.85
$\sigma =$ 160	PSNR	37.65	37.75	37.69	37.70
	SSIM	0.83	0.82	0.83	0.83
$\sigma =$ 180	PSNR	37.18	37.27	37.23	37.23
	SSIM	0.80	0.79	0.80	0.80
$\sigma =$ 200	PSNR	36.74	36.82	36.79	36.79
	SSIM	0.77	0.76	0.77	0.77
$\sigma =$ 220	PSNR	36.33	36.41	36.39	36.38
	SSIM	0.74	0.73	0.74	0.74
$\sigma =$ 240	PSNR	35.95	36.04	36.01	36.00
	SSIM	0.71	0.70	0.71	0.71
		DDAE Model			
		1 $^{\mathbf {st}}$5-fold	2 $^{\mathbf {nd}}$5-fold	3 $^{\mathbf {rd}}$5-fold	average
$\mathbf {m}=8$	PSNR	28.72	28.56	28.53	28.60
	SSIM	0.89	0.88	0.89	0.89
$\mathbf {m}=16$	PSNR	28.58	28.39	28.51	28.49
	SSIM	0.89	0.88	0.89	0.88
$\mathbf {m}=32$	PSNR	28.57	29.08	28.92	28.86
	SSIM	0.89	0.90	0.89	0.89

Note: Yellow colors highlight the optimal PSNR for each 5-fold. Orange colors highlight the optimal average values of PSNR and its corresponding SSIM. For Gaussian filter, median filter, and BM3D, the optimal sigma value is chosen based on the average PSNR.

### B. Denoising of Third Harmonic Generation Images

**FIGURE 5.**
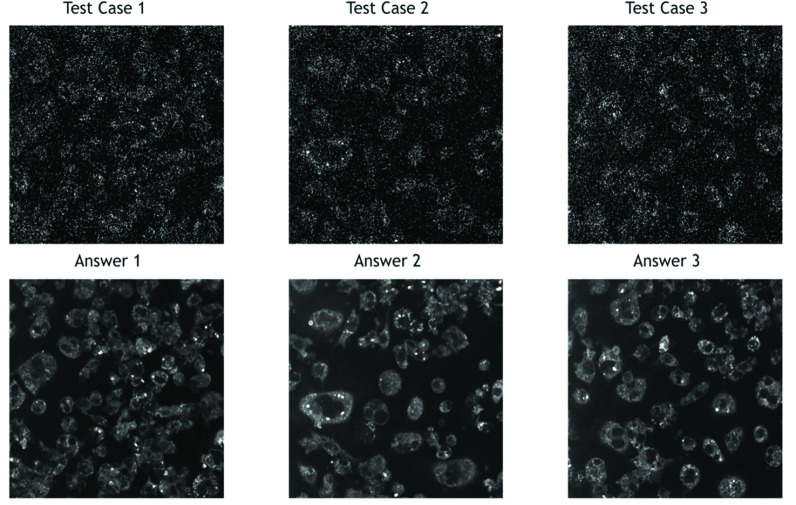
Noisy inputs (upper rows) and low-noise answers (bottom rows) of three testing THG images of RAW cells. Fields of views: $160\times 160\,\,\mu \text{m}$.

**FIGURE 6.**
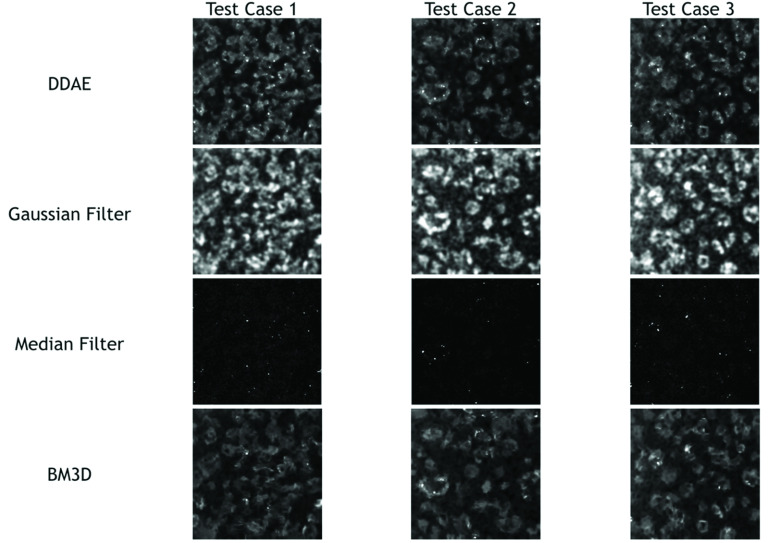
Denoising results of THG images with DDAE, Gaussian filter, median filter, and BM3D algorithms.

Similarly, we built THG DDAE models by applying 31 batches to 5-fold cross-validation, composed of 200 THG images acquired at 31 different locations on the petri-dish. Instead of the nuclei, the THG images revealed granules in the cytoplasm (Fig. 5), which delineated the outline of the cells. The results (Fig. 6) show that the THG DDAE model also outperforms

most traditional methods in terms of both the PSNR and SSIM scores (Table 2 and Appendix Table 5), except BM3D. In addition, among all of the denoising methods, the PSNR of BM3D and the SSIM of Gaussian filter are the best, which achieve 26.38 and 0.52, respectively. The BM3D method performs relatively well in terms of its signal-to-noise ratio, but becomes vague at the cellular boundaries. In the Gaussian filter, we find that it can retain the general structure of cells, but there is still significant noise in the background. Similar to the case in 3PF images, the median filter retains the structures and signals of the cells. In general, the THG DDAE model was the better choice for denoising THG microscopy images in terms of PSNR ( $=21.66$) and SSIM ( $=0.70$).

**TABLE 5:** Denoising Performance of Filters on THG Images

		Gaussian Filter			
		1 $^{\mathbf {st}}$5-fold	2 $^{\mathbf {nd}}$5-fold	3 $^{\mathbf {rd}}$5-fold	average
$\sigma =$ 1	PSNR	16.13	16.33	16.42	16.29
	SSIM	0.34	0.34	0.35	0.34
$\sigma =$ 3	PSNR	16.43	16.64	16.74	16.60
	SSIM	0.46	0.47	0.47	0.47
$\sigma =$ 5	PSNR	16.25	16.45	16.55	16.41
	SSIM	0.48	0.49	0.50	0.49
$\sigma =$ 10	PSNR	15.88	16.08	16.18	16.05
	SSIM	0.51	0.52	0.52	0.52
		Median Filter			
		1 $^{\mathbf {st}}$5-fold	2 $^{\mathbf {nd}}$5-fold	3 $^{\mathbf {rd}}$5-fold	average
$\sigma =$ 1	PSNR	12.68	12.86	12.91	12.82
	SSIM	0.15	0.16	0.16	0.16
$\sigma =$ 3	PSNR	13.95	14.16	14.25	14.12
	SSIM	0.12	0.12	0.12	0.12
$\sigma =$ 5	PSNR	13.79	14.00	14.09	13.96
	SSIM	0.10	0.11	0.11	0.10
$\sigma =$ 10	PSNR	13.71	13.92	14.01	13.88
	SSIM	0.09	0.10	0.10	0.10
		BM3D			
		1 $^{\mathbf {st}}$5-fold	2 $^{\mathbf {nd}}$5-fold	3 $^{\mathbf {rd}}$5-fold	average
$\sigma =$ 120	PSNR	26.21	26.41	26.51	26.38
	SSIM	0.40	0.41	0.41	0.41
$\sigma =$ 140	PSNR	26.04	26.25	26.34	26.21
	SSIM	0.38	0.38	0.38	0.38
$\sigma =$ 160	PSNR	25.89	26.10	26.19	26.06
	SSIM	0.36	0.36	0.36	0.36
$\sigma =$ 180	PSNR	25.76	25.97	26.06	25.93
	SSIM	0.34	0.34	0.34	0.34
$\sigma =$ 200	PSNR	25.65	25.85	25.94	25.81
	SSIM	0.32	0.33	0.33	0.33
$\sigma =$ 220	PSNR	25.54	25.75	25.84	25.71
	SSIM	0.31	0.32	0.31	0.31
$\sigma =$ 240	PSNR	25.45	25.66	25.75	25.62
	SSIM	0.30	0.30	0.30	0.30
		DDAE Model			
		1 $^{\mathbf {st}}$5-fold	2 $^{\mathbf {nd}}$5-fold	3 $^{\mathbf {rd}}$5-fold	average
$\mathbf {m}=8$	PSNR	21.53	21.68	21.79	21.66
	SSIM	0.69	0.70	0.70	0.70
$\mathbf {m}=16$	PSNR	21.54	21.65	21.80	21.66
	SSIM	0.69	0.70	0.71	0.70
$\mathbf {m}=32$	PSNR	21.52	21.65	21.70	21.62
	SSIM	0.69	0.70	0.70	0.70

Note: Yellow colors highlight the optimal PSNR for each 5-fold. Orange colors highlight the optimal average values of PSNR and its corresponding SSIM. For Gaussian filter, median filter, and BM3D, the optimal sigma value is chosen based on the average PSNR.

### C. Preservation of Nuclear and Cellular Boundaries

**FIGURE 7.**
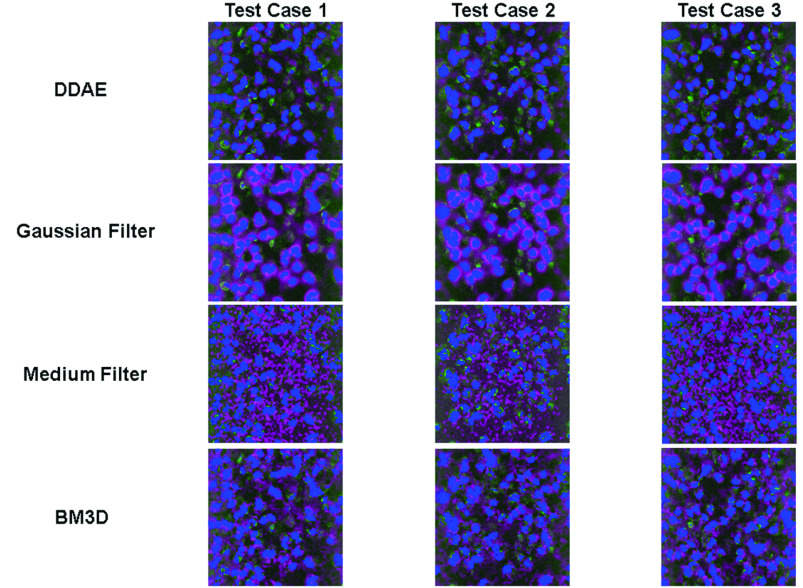
Nuclear region analysis of 3PF images denoised with DDAE, Gaussian filter ( $\sigma =5$), median filter ( $\sigma =10$), and BM3D ( $\sigma =240$) algorithms. Blue: true positive, Magenta: false positive, Green: false negative.

**FIGURE 8.**
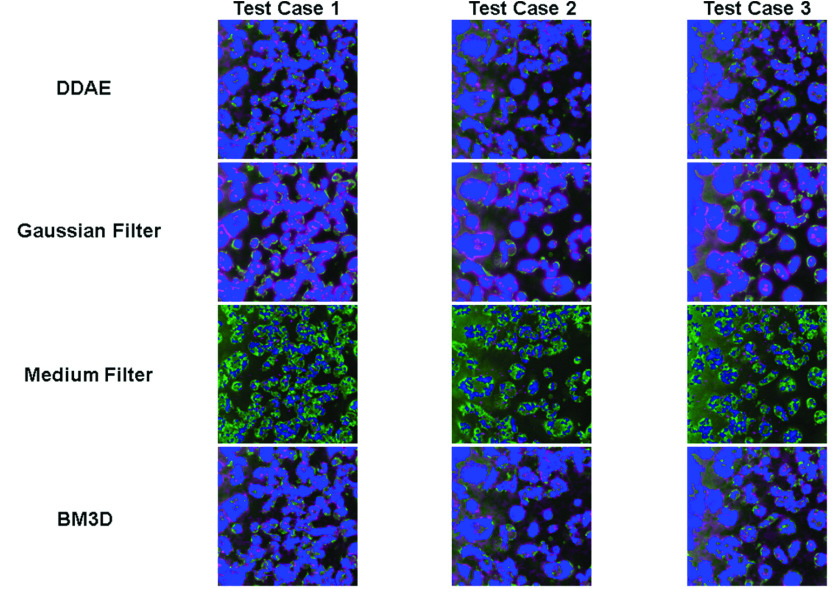
Cellular region analysis of THG images denoised with DDAE, Gaussian filter ( $\sigma =5$), median filter ( $\sigma =5$), and BM3D ( $\sigma =180$) algorithms. Blue: true positive, Magenta: false positive, Green: false negative.

To understand whether the DDAE can preserve more stochastic resonance-enhanced signals than the other denoising filters, following the procedures described in the Methods section, we identified pixels that represent the regions of nuclei or cells in both denoised and low-noise answer images. We then computed their precision, recall rates, and F1 scores (Table 3 and Appendix Table 5) to evaluate how well the boundary information was preserved after denoising (Figs. 7 and 8). For the 3PF cases, we found that the 58-59% precision rate of the DDAE mode is higher than that of most of the filters, except for a few cases of median filters ( $\sigma =$ 3 or 5) in extreme situations that have very low recall rates of 8% and 10%. The 76-78% recall rate of DDAE performed better than most BM3D and median filters but not as good as Gaussian Filters (84–91%). This indicates that DDAE generates more false negatives (green in Fig. 7) than BM3D in the analysis of nuclei boundaries. Balanced with the F1-score, which takes precision and recall as equal weighting, the DDAE16 model has an average score of 0.667, which is much higher than that of the other filters. For the THG cases, the signal level and dynamic range of the low-noise answer image were much lower than those of 3PF. We obtained an average 65-66% precision rate for DDAE, which is lower than part of the median filter results. However, DDAE’s average 83% recall rate is higher than theirs. This result indicates that DDAE may result in more false-positive pixels and fewer false-negative pixels in the THG images of the cytoplasm. Balanced with the F1-score, the 0.736 scores of the DDAE model again outperformed the other filters. These results indicate that for low photon-budget images containing stochastic resonance-enhanced signals, DDAE can retain nuclear or cellular boundaries more accurately for further segmentation.

## IV. Discussion and Conclusion

For low photon-budget multiphoton biomedical imaging, it is crucial to find a balanced denoising method to preserve stochastic resonance-enhanced regions and retain cell boundary features for further segmentation. The despeckle denoising strategy removes most of the noise-boosted signals and corrodes the cell regions, whereas the low-pass spatial filtering strategy sacrifices resolution and expands cellular regions. Nonlocal mean algorithms such as BM3D and Gaussian filters average the patches with similar textures and achieve state-of-the-art performance; however, these methods involve either time-consuming optimization processes or manually chosen parameters, which result in low computational efficiency when pursuing high performance. This problem becomes an analysis bottleneck for many high-throughput 3D microscopies.

Our results for the compact DDAE model revealed its capability to realize both feature preservation and noise filtering. After several epochs of training, the validation loss was significantly reduced (Appendix Fig. 9 and 10). The optimization and parameter selection process can be accomplished in advance during the training of the DDAE model, and the denoising process requires only a few seconds. Even at such a high denoising speed, the DDAE model outperformed other conventional methods in preserving signal regions but sacrificing a bit of denoising ability in PSNR. It shows that DDAE under low-phonon budget conditions is a better trade-off approach between structural similarity and the preservation of the nuclear or cellular boundaries. This may be owed to the nonlinear transformation characteristics of deep learning. The stochastic noise in low photon-budget multiphoton imaging is not a perturbative interference to signals. It can nonlinearly boost subthreshold signals above the detection limit. Hence, methods such as Gaussian filters and BM3D may be unable to handle such a situation well within a short computation time. In contrast, the deep learning model could learn the nonlinear features from the training dataset, effectively suppress the noise, and correctly preserve the cellular boundaries. This pilot study explored the effectiveness of the DDAE system for boundary-preserved image denoising. We plan to explore advanced deep learning models to further improve denoising performance.

**FIGURE 9.**
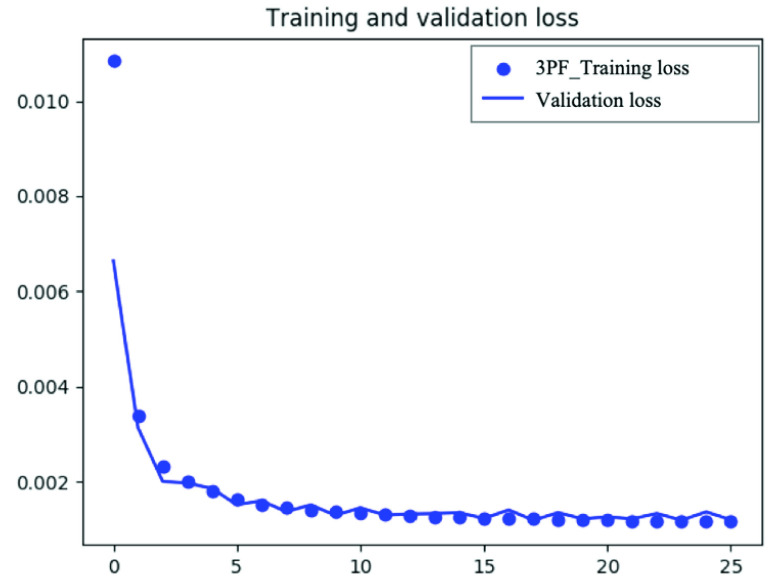
Training and validation loss across different epoch iteration during the DDAE training of 3PF images.

**FIGURE 10.**
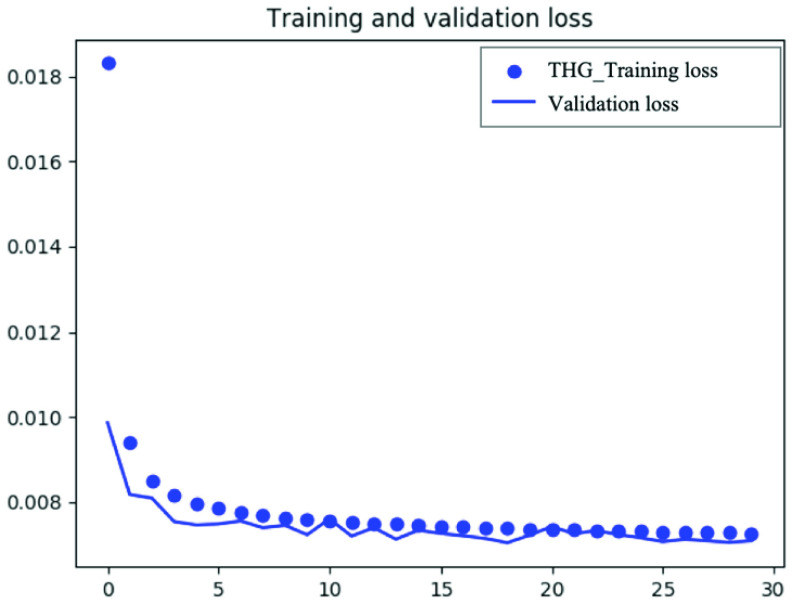
Training and validation loss across different epoch iteration during the DDAE training of THG images.

This study also investigated the effects of three filter sizes ( $m=8$, 16, and 32) on the denoising performance of DDAE. The results show that the performance of DDAE models with different filter sizes varies while using different images and evaluation metrics. For example, DDAE with $m=32$ achieves the best PSNR and SSIM in 3PF images but has relatively limited performance in THG images (Appendix Table 4 and 5). However, the differences between DDAE models are extremely low (< 1%). It shows the proposed DDAE model with smaller filter sizes ( $m=8$) requiring less computation complexity is sufficient and more suitable to enhance image quality and preserve nuclear and cellular boundaries.

Previous studies have demonstrated the need for effective image denoising tools in clinical applications. For example, [14] white blood cell counting is a routine practice for medical diagnosis, which requires high-SNR white blood cell images of size, shape, structure, and nucleus-to-cytoplasm ratio to accurately classify and identify cells based on morphological phenotypes. The proposed DDAE could restore nuclear and cellular boundaries, which supports nucleus segmentation in white blood cell counting [43]. Texture analysis after segmentation could further improve the accuracy and reliability of white blood cell counting.

In future works, because the DDAE model has the potential to improve image quality from various types of image sources, we plan to implement our DDAE model on different types of microscopy images for practical medical technology applications. To reduce the operation time, we plan to develop a lightweight DDAE using advanced machine-learning techniques at a low photon budget condition and under limited hardware resources, such as by using pruning and tinyML. In addition, Lehtinen *et al.* showed that without clean dataset, the deep learning model can still perform good image restoration [44]. We will also attempt to train the neural network with cleaner images excited by a higher laser power and detected at a lower gain. A reliable denoising method can speed up applications, such as high-throughput image segmentation, fast 3D morphodynamic analysis, and long-term cell tracking. We expect that this denoising method can be organically integrated into the process of imaging analysis, help improve the processing efficiency of many high-throughput microscopies, and achieve online denoising on hardware.

## Appendix

See Figures 9 and 10 and Tables 4 and 5.
